# NAMPT serum levels are selectively elevated in acute infectious disease and in acute relapse of chronic inflammatory diseases in children

**DOI:** 10.1371/journal.pone.0183027

**Published:** 2017-08-24

**Authors:** Julia Gesing, Kathrin Scheuermann, Isabel Viola Wagner, Dennis Löffler, Daniela Friebe, Wieland Kiess, Volker Schuster, Antje Körner

**Affiliations:** 1 Hospital for Children and Adolescents, University Leipzig, Leipzig, Saxony, Germany; 2 Leipzig University Medical Center (IFB) AdiposityDiseases, Leipzig, Saxony, Germany; 3 Hospital for children and adolescents, University Hospital Cologne, Cologne, North Rhine-Westphalia, Germany; 4 Fraunhofer Institute for Cell Therapy and Immunology Leipzig, Leipzig, Saxony, Germany; University College London, UNITED KINGDOM

## Abstract

Nicotinamide phosphoribosyl transferase (NAMPT) is an inflammatory adipocytokine shown to interact in immune modulation in chronic inflammatory diseases, acute respiratory distress syndrome, sepsis, cancer and obesity in adulthood. It is, however, not clear whether this association reflects a chronic elevation or acute inflammatory response. We analyzed NAMPT concentrations in distinct states of inflammation in 102 children and found consistently significantly increased NAMPT levels in subjects with acute infections. NAMPT concentrations in children with stable chronic inflammatory diseases were not significantly different, whereas in patients with acute relapse of chronic disease NAMPT was significantly higher than in children in remission or healthy controls. In states of low-grade inflammation (children with atopic disease or obesity) we did not detect alterations in NAMPT serum levels. NAMPT correlated positively with inflammatory markers such as CRP. The most predictive factor for NAMPT serum concentrations was leucocyte count and therein the neutrophil count. Furthermore, systemic circulating NAMPT levels were closely associated with NAMPT release from corresponding cultured PBMCs. In conclusion, NAMPT is selectively increased in states of acute but not chronic inflammation in children. The close relationship between systemic circulating NAMPT with leucocyte counts and release indicate that leucocytes most probably are the source of inflammation related NAMPT levels.

## Introduction

Nicotinamide Phosphoribosyltransferase (NAMPT), also known as Pre-B-cell colony-enhancing factor (PBEF) or visfatin was shown to play a prominent role in cell metabolism, inflammation and immune modulation [1,2]. NAMPT was originally isolated from a cDNA library of human peripheral blood lymphocytes [3] acting as a growth factor for B-cell development. Later, intracellular NAMPT was found to act as the rate-limiting enzyme in the salvage pathway restoring the cofactor nicotinamide adenine dinucleotide (NAD) in mammals [4]. Recently a knock-out mouse-model showed NAMPT to be an essential gene for survival [5].

In obesity research NAMPT (in this context called visfatin) gained attention as an adipokine [6,7], although its function remains controversial with regard to obesity and glucose metabolism with positive, negative or no associations found [8]. NAMPT variants were associated to childhood obesity in an Indian cohort [9] but not a danish cohort [10]. We have previously shown that the elevated enzymatically active NAMPT levels in obese children are mainly derived from leucocytes and inducible by lipopolysaccharide and hence that NAMPT may serve as a biomarker or even mediator linking obesity, inflammation and insulin resistance [11]. Observations that the highest expression of NAMPT in the human body was found in leucocytes [12], that extracellular NAMPT increases the production of inflammatory cytokines (IL-8, IL6, TNFalpha, IL-1ß) [13] and that NAMPT inhibits neutrophil apoptosis in experimental inflammation and clinical sepsis [14] and activates T cells, B cells and monocytes [13] point to a role for NAMPT in inflammation and inflammatory disease. In line with this, NAMPT was shown to be a marker of chronic diseases like inflammatory bowel diseases [15] or rheumatoid arthritis [16] further suggesting that NAMPT is involved in the regulation of inflammation.

There are only few studies focussing on NAMPT in inflammatory states during childhood [17,18]. We have evidence indicating that NAMPT is elevated in childhood obesity and its corresponding low-grade inflammation [12]. In this study we aimed to focus on NAMPT in distinct entities of inflammatory processes during childhood and adolescence. For this, we evaluated NAMPT levels in children and adolescents with acute infections, chronic inflammatory diseases (active and inactive), obesity and atopic diseases in comparison to a healthy control group.

## Research design and methods

### Patient cohort

We recruited 102 children and adolescents between 0 and 18 years from our inpatient and outpatient units of the University Children´s Hospital Leipzig into this study. Exclusion criteria were oncological, syndromal diseases, and non-inflammatory chronic diseases. Written informed consent was obtained from the legal representatives and children older than 12 years. The study was approved by the ethic committee of the University of Leipzig (Reg. No: 357-10-1312200). Anthropometric data, gender, medical history and medication were documented. We collected serum and EDTA blood samples during clinically indicated venous punctures.

Patients were stratified into four study groups encompassing acute inflammatory states, chronic inflammatory diseases, atopic/allergic manifestations and obese patients (Table 1). The healthy control group consisted of lean and healthy children and adolescents. Obese patients with no other diagnosis were analyzed as their own group. Anthropometric data and the inflammatory profile of each study group are given in Table 2. Mean age was lower in the acute group, whereas naturally anthropometric parameters were higher in the obese group, and also in the atopic group (Table 2 and S1 Table).

**Table 1:** Clinical diagnosis of probands in the study groups.

Acute (n = 16)		Chronic* (n = 36)		Allergic (n = 16)	
	n		n (a)		n
■ Acute bronchitis ■ Acute pneumonia ■ Upper respiratory tract infection ■ Acute gastroenteritis ■ Kawasaki disease ■ Mononucleosis ■ Acute Otitis media ■ Acute Tonsillitis	3 3 3 2 2 1 1 1	■ Cystic fibrosis ■ Juvenile idiopatic arthritis (JIA) ■ Chronic inflammatory bowel disease ■ Chronic recurrent multifocal osteomyelitis (CRMO) ■ Isolated chronic uveitis ■ Reactive / unspecified arthritis ■ Chronic otitis ■ Lyme arthritis ■ Intestinal tuberculosis ■ Chronic tenosynovitis ■ Systemic lupus erythematodes	7 (2) 7 (3) 5 (2) 4 3 (1) 3 (2) 2 (2) 2 (1) 1 (1) 1 (1) 1	■ Asthma ■ Atopic dermatitis ■ Hay fever ■ Multiple allergies ■ Urticaria	9 3 2 1 1

n Number of patients with the same diagnosis. (a) Number of patients with symptomatic chronic disease at the time of sample collection.

* Patients from the chronic group received standard treatment of their underlying disease. 13 patients received steroid and/or immunmodulating medication. 10 Patients received non-steroidal anti-inflammatory medication.

**Table 2:** Characterization of the cohort (n = 102).

	Control (n = 15)		Acute (n = 16)		Chronic (n = 36)		Allergic (n = 16)		Obese (n = 19)	
Parameters	Mean ± SD	*(range)*	Mean ± SD	*(range)*	Mean ± SD	*(range)*	Mean ± SD	*(range)*	Mean ± SD	*(range)*
**Anthropometric**										
Male / Female	10/5		13/3		11/25		11/5		10/9	
Age (years)	11.93 ±5.35	0.92–17.9	**5.27 ±4.81**	0.37–16.68	10.8 ±4.21	0.51–16.6	11.08 ±2.7	5.63–5.08	12.97 ±3.03	5.8–17.93
Pubertal stage	1.56 ±0.73	1–3	1 ±0	1	1.29 ±0.46	1–2	1.4 ±0.74	1–3	2.26 ±0.73	1–3
Height SDS_LMS_	-1.09 ±1.46	-4.3	0.18 ±1.15	-4.45	-0.23 ±1.1	-5.23	0.11 ±1.61	-6.14	**0.67 ±1.39**	-5.39
BMI (kg/m^2^)	18.6 ±2.57	14.154–22.1	17.61 ±3.3	13.16–23.98	18.05 ±3.41	12.65–28.69	**24.73 ±8.19**	13.08–41.5	**31.43±6.04**	24.37–0.65
BMI SDS_LMS_	- 0.47±0.66	-3.32–0.98	0.57±1.26	-0.97–2.78	- 0.008±1.17	-2.04–2.22	**1.42±1.69**	-2.8–3.48	**2.84±0.77**	1.41–4.6
Lean (BMI < 1.22 SDS_LMS_)	15		13		31		5		0	
**Inflammatory**										
CRP (mg/l)	1.63 ±2.49	0.15–6.58	21.2 ±49.9	0.38–200.13	1.57 ±2.11	0.15–7.48	N/A		N/A	
ESR 1h (mm)	7.2 ±3.55	2–13	**42 ±5.94**	37–50	15.14 ±9.55	2–37	16.86±13.13	7–45	18.77 ±19.85	2–70
Leucocyte count (/nl)	6.42 ± 1.92	3.8–10.6	**9.52 ±2.79**	5.1–15.6	8.47 ±4.15	3.5–21.1	5.78 ±1.23	4.3–8.6	6 ±1.4	4.4–9.6
Lymphocytes (/nl)	2.6 ±1.42	1.51–6.69	3.01±1.72	1.08–6.55	2.87 ±2.57	0.62–15.81	2.03 ±0.49	1.3–3.06	2.19 ±0.78	1.12–4.11
Monocytes (/nl)	0.54 ±0.17	0.26–0.88	**1.06 ±0.67**	0.32–2.69	0.66 ±0.36	0.16–1.83	0.55 ±0.17	0.31–0.91	0.49 ±0.11	0.21–0.64
Neutrophil Granulocytes (/nl)	2.91 ±1.05	0.96–4.69	5.13 ±3.47	0.63–12.8	4.73 ±2.97	0.16–1.83	2.97 ±0.84	2.02–4.63	3.12 ±1.15	1.87–6.52
Basophil Granulocytes (/nl)	0.04 ±0.03	0.01–0.12	**0.09 ±0.07**	0.02–0.06	0.03 ±0.04	0–0.21	0.02±0.01	0.01–0.04	0.02 ±0.01	0.01–0.05
Eosinophil Granulocytes (/nl)	0.32 ±0.43	0.06–1.7	**0.15 ±0.21**	0–0.73	0.17 ±0.14	0–0.63	0.2 ±0.09	0.06–0.34	0.17 ±0.17	0.05–0.64

BMI, body mass index; CRP, C-reactive protein; ESR, erythrocyte sedimentation rate; SDS_LMS_, standard deviation score; Pubertal stage (1 = prepubertal; 2 = pubertal, 3 = postpubertal). Significant *p-*values (*p*<0.05) are indicated in **bold**. Non-normally distributed data was log-transformed before analysis.

### Cultivation of peripheral blood mononuclear cells (PBMC)

After collection of EDTA samples leucocytes were separated by centrifugation after lysis of erythrocytes with lysis buffer (8.29 g NH_4_Cl; 1 g KHCO_3_; 0,0372 g Na-EDTA in 1 l H_2_O; pH 7.29). Leucocytes were incubated in RPMI medium containing 0.5% FCS, penicillin and streptomycin at 37°C for one hour before harvesting of cells and supernatant fractions. Cells could be harvested and supernatants obtained in 80% of controls, 50% of patients with acute infections, 64% of patients with chronic disease, 100% of patients with allergies and 95% of obese subjects.

### Quantification of NAMPT in serum samples and supernatants

NAMPT concentrations were measured in serum samples and supernatant fractions by ELISA following the manufacturer’s protocol (Adipogen, Seoul, South Korea). Assay quality variables including sensitivity and specificity have been validated as published before [12].

### Analyses of inflammatory markers

Inflammatory markers were assessed by a certified laboratory (Institute of Laboratory medicine, clinical chemistry and molecular diagnostics Leipzig, Germany) applying routine diagnostic procedures. The C-reactive protein (CRP) was measured with the CRPL3 immunoturbidimetric assay (Roche Diagnostics GmbH) on Roche/Hitachi cobas systems. The hemogram was assessed with automated blood count using laser flow cytometry, electrical impedance and cyanide-free sodium lauryl sulphate methods on a Sysmex XE2100.

### Statistical analyses

Logarithmic transformation of non-normally distributed data was performed before analysis. For comparison of quantitative traits between groups, oneway ANOVA was applied. Correlation analyses were performed using Pearson correlation analysis or partial correlation analysis with adjustment for height SDS_LMS_ (standard deviation score using the LMS values by Kromeyer-Hausschild [19]) and neutrophil granulocyte count. For multiple regression analyses the stepwise forward model was used. For all tests, the significance level was set at 0.05. Statistical analyses were performed using the software package Statistica 7.1 (StatSoft, Tulsa, OK, USA) and GraphPad Prism5.

## Results

### NAMPT serum concentrations are increased in acute states of inflammation

**Fig 1:**
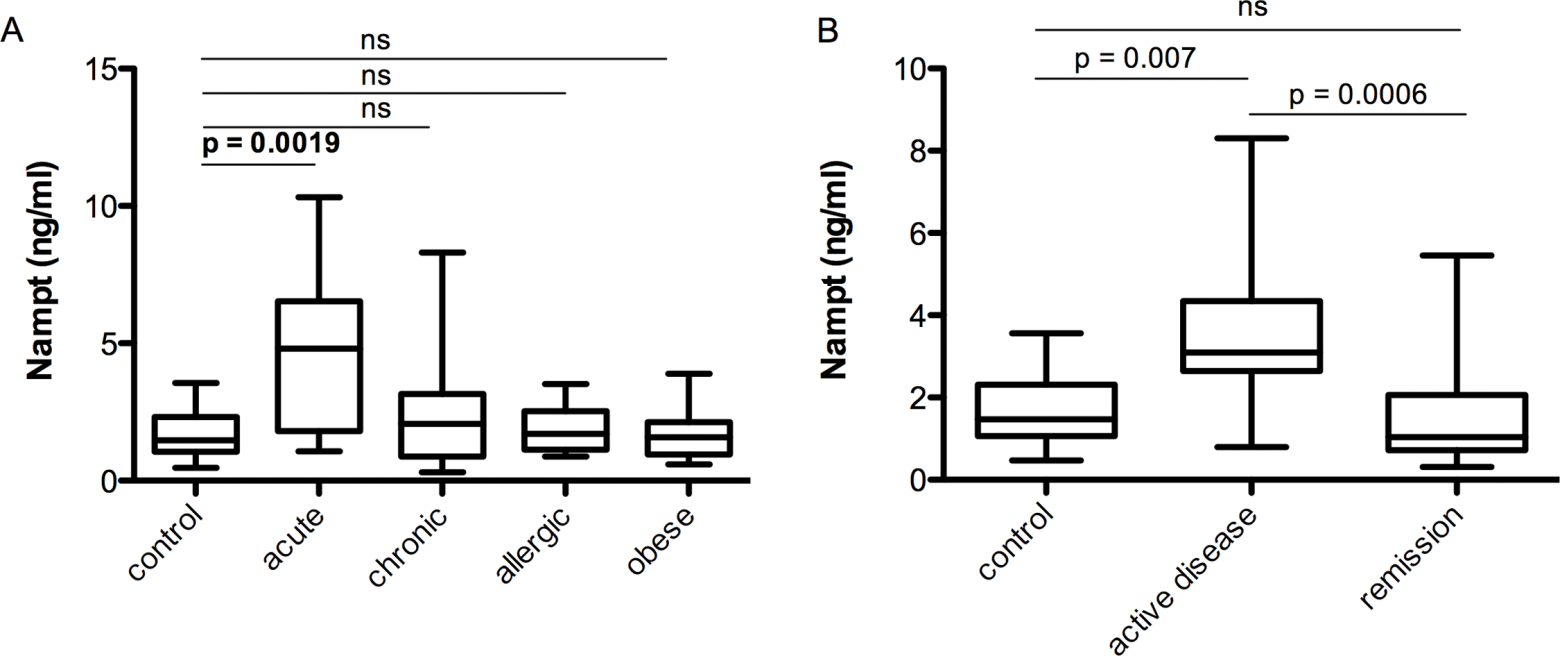
NAMPT serum concentrations in distinct inflammatory conditions. Comparison of NAMPT concentrations between patient groups of distinct inflammatory conditions and controls (A). Patients with chronic inflammation were further stratified into patients with active disease vs. patients without symptoms (remission) (B). Boxes are interquartile range, whiskers are minimum to maximum. Statistical significance was assessed by ANOVA and Tukey HSD test in log transformed values.

To evaluate whether NAMPT serum concentrations are associated with acute or chronic inflammation, atopic status, or obesity, we measured NAMPT in serum samples in our cohort. We found highest NAMPT levels in the acute group compared to the healthy controls and all other study groups (Fig 1A). No difference was found in NAMPT serum concentrations between patients with chronic inflammatory diseases, allergic manifestations or obesity compared to the healthy lean controls.

Considering that disease activity may differ in patients with chronic inflammatory diseases we compared NAMPT serum concentrations of patients with active chronic disease or relapse of clinical symptoms to patients in remission and healthy controls. We found significantly higher NAMPT serum concentrations in the subgroup with symptomatic chronic disease compared to patients in remission (Fig 1B).

Hence, overall NAMPT is increased in acute but not in chronic states of inflammation or infection.

### NAMPT serum concentrations are associated with markers of inflammation

**Fig 2:**
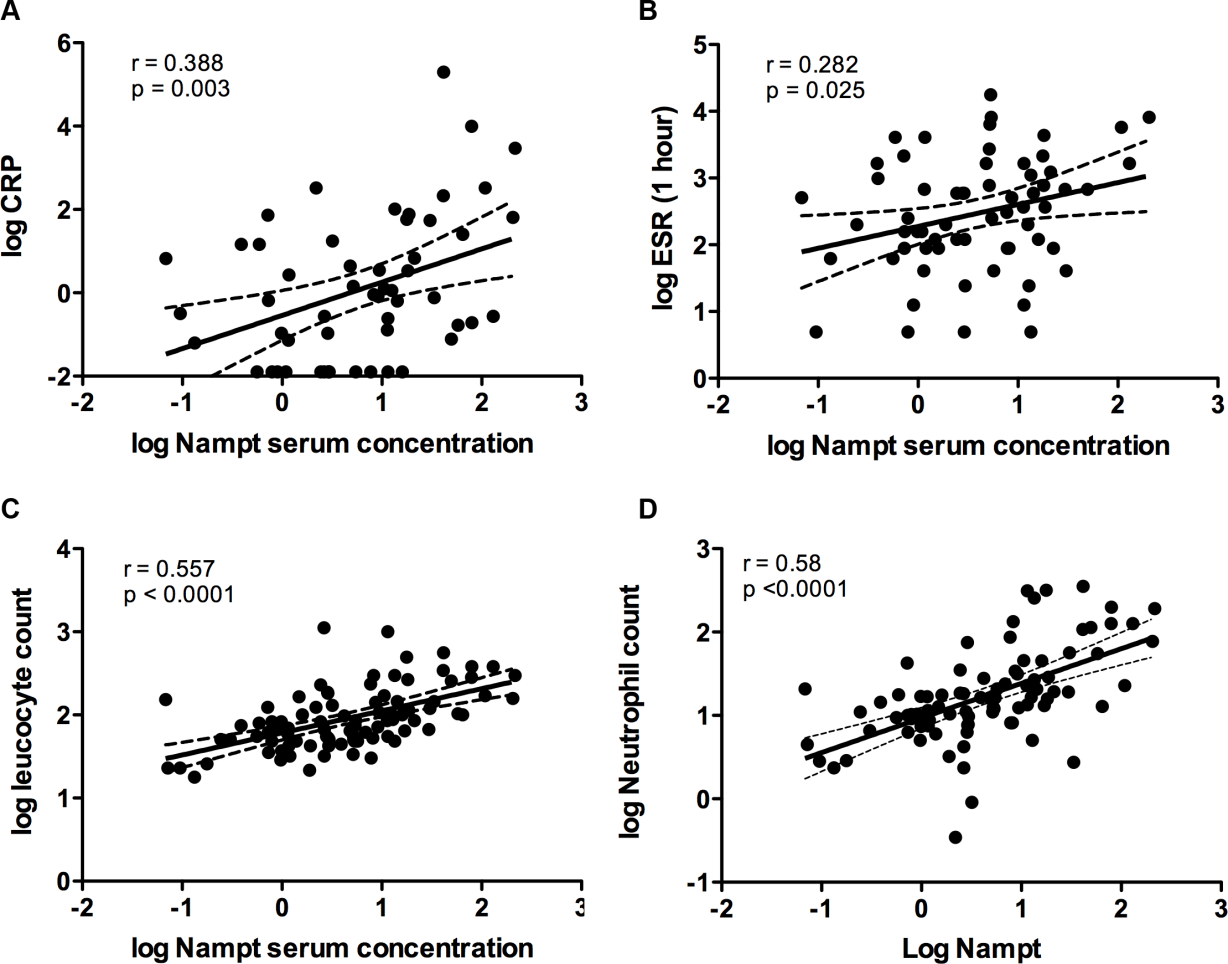
Positive correlation between NAMPT serum levels and inflammatory markers. Correlation between log NAMPT serum concentration and log C-reactive protein (A), log ESR (1 hour) (B), log leucocyte count(C) and log neutrophil granulocyte count (D).

Considering our results on selectively increased NAMPT levels in acute inflammation, we next analyzed the association of NAMPT with common inflammatory markers. NAMPT serum concentrations correlated positively with CRP and the erythrocyte sedimentation rate (ESR) (Fig 2A and 2B). We did, however, find highest correlation of NAMPT serum levels with the leucocyte count (Fig 2C). In a general linear model including age, patient study group and the inflammatory parameters ESR, CRP and leucocyte count, the leucocyte count solely predicted (log) NAMPT serum levels (β = 0.679, p = 0.002), hence independent from the pathologic origin of inflammation.

Differential analyses on leucocyte subspecies revealed the highest correlation of NAMPT with neutrophil granulocyte count (Fig 2D) and also to some degree with monocyte count (r = 0.24, p = 0.022), whereas there was no relationship with lymphocytes, basophil or eosinophil granulocyte counts.

Hence, the inflammation associated increase in NAMPT serum levels was most closely related to neutrophil granulocyte counts.

### Systemic NAMPT levels are related to NAMPT release from corresponding PBMCs

**Fig 3:**
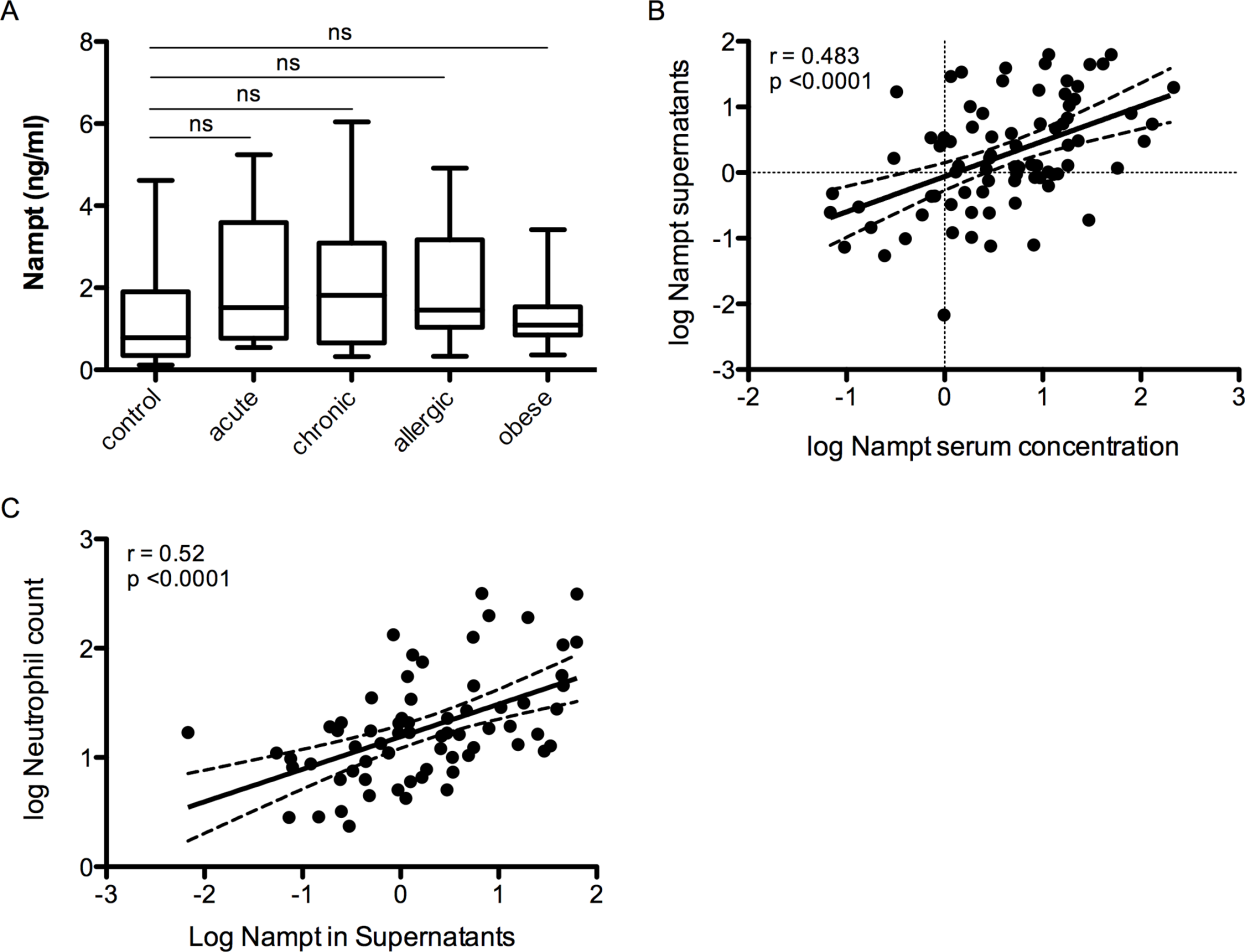
NAMPT release from PBMCs. Comparison of NAMPT release between patient groups of distinct inflammatory conditions and controls (A). Boxes are interquartile range, whiskers are minimum to maximum. Statistical significance was assessed by ANOVA and Tukey HSD test. Correlation between log NAMPT serum concentrations and corresponding NAMPT release from PBMCs of the same patient (B) and Correlation between NAMPT release from PBMCs with blood neutrophil counts (C).

To assess whether leucocytes directly contribute to inflammatory associated NAMPT increase, we evaluated NAMPT release into supernatants of peripheral blood mononuclear cells (PBMCs) of our patients. We found no significant difference between the patient groups (Fig 3A). But NAMPT release from PBMCs correlated closely with NAMPT serum levels (Fig 3B), particularly with the neutrophil granulocyte subset (Fig 3C).

Hence, the NAMPT production and release by leucocytes appears to underline the association with inflammatory markers in states of acute inflammation.

## Discussion

In this study we show that circulating levels of NAMPT are selectively increased in children and adolescents with acute infections and symptomatic chronic inflammatory conditions compared to healthy controls and also compared to patients with asymptomatic chronic inflammatory diseases, allergic manifestations or obesity. We further show that the increased NAMPT serum levels correlated with inflammatory parameters and most closely with blood leucocyte counts, particularly neutrophils, and indeed release of NAMPT protein from PBMC was related to systemic serum NAMPT levels.

Only few studies analyzed NAMPT in the context of inflammatory processes in childhood [17,18]. We had previously shown, that mainly mono- and granulocytes secrete circulating NAMPT and that mildly elevated leucocytes in obesity may contribute to the phenomenon of slightly increased NAMPT levels in low-grade inflammation in obesity [12]. We, therefore, were interested whether NAMPT may serve as an inflammatory parameter in other types of inflammation. Here, we found that the increase in NAMPT with inflammation is selective for acute inflammation as in infections or in acute relapse of chronic inflammation, also in comparison to other, more subtle types such as atopic disease or chronic inflammation. We suppose the increased NAMPT serum concentration in acute inflammatory response and relapse of chronic inflammation is due to its release from neutrophil granulocytes. Naturally, there is leucocytosis with or without relative neutrophilia in acute inflammation or monocytosis in chronic inflammation.

One may assume that the release of extracellular NAMPT is triggered by inflammation markers in inflammatory conditions. If this was the case we could assume that activated granulocytes release more NAMPT than resting granulocytes. We therefore evaluated NAMPT release into supernatants of cultured PBMCs of our patients, which did not differ significantly between different inflammatory states and the controls. Limitations were the small number of PBMC cultivation and the heterogenous groups. Still we see a positive correlation of NAMPT release to the number of neutrophils in the blood count and also to NAMPT serum concentrations.

Comparing all patients with chronic inflammatory diseases to the control group did not show significant differences in NAMPT serum levels. Only, when we compared the subgroup of patients with symptomatic chronic disease to patients in remission or the control group, significantly higher NAMPT serum concentrations, comparable to those in patients with acute infections were detected. This is partly in line with reports where NAMPT was found to be a marker of chronic inflammation [20] in adults and correlated positively to disease activity in inflammatory bowel disease [21], rheumatoid arthritis [22], Graves' disease with association with autoantibody titer [23] or also in local inflammatory disease of periodonditis [24]. Furthermore, microbial and inflammatory signals such as Il-1β were shown to locally enhance NAMPT synthesis [25]. It is, however, not clear whether this association reflects a chronic elevation in persisting inflammation or is limited to acute inflammatory response. We extend these findings by showing that in our pediatric cohort particularly in chronic active disease, but not as strong in chronic stable disease, NAMPT serum levels were elevated. We speculate that NAMPT could serve as a marker for disease activity of chronic inflammatory disease and severity of inflammation status. This is in line with one previous report where NAMPT was increased in acute pneumonia and associated with severity and leucocyte count [26]. Also in neonatal sepsis NAMPT was found to be significantly elevated [18]. NAMPT was a predictor of mortality in sepsis and pneumonia [26,27].

The majority of patients with chronic diseases were under treatment with non-steroidal anti-inflammatory or immune-modulating medication. We cannot exclude that NAMPT levels were altered by this treatment. Still, we detected the highest NAMPT level in the chronic group (8.3 ng/ml) in a patient with Crohn’s disease under immune-modulating therapy. We can assume that no matter if there is an effect of the medication to NAMPT levels, higher levels indicate higher disease activity. This could be of value in the treatment of children with chronic inflammatory diseases.

The mechanism by which NAMPT interacts with the immune response in not fully clear yet. NAMPT catalyzes the rate-limiting step in the salvage pathway for NAD(+) biosynthesis, and thereby regulates the deacetylase activity of sirtuins [8]. This NAMPT-NAD(+)-SIRT axis has been postulated in articular chondrocytes to be involved in cartilage destruction in osteoarthritis [28]. NAMPT was further reported to induce lung NFkappaB transcriptional activities and inflammatory injury via direct ligation of Toll-like receptor 4 (TLR4) [29]. A deeper insight into these mechanisms may also provide new therapeutic strategies in inflammatory disease. Even beyond the mere clinical association with inflammatory disease such as juvenile idiopathic arthritis, NAMPT was found to inhibit the pharmacological activity of methotrexate and was suggested as a predictive biomarker of response, as well as a potential therapeutic target [30]. NAMPT inhibition with FK866 has anti-inflammatory activity in different models of immune disorders through lowering the production of neutrophil chemoattractants [1]. NAMPT blockade also had beneficial effects in an acute lung injury model and may modify the cancer microenvironment through their anti-inflammatory properties [31]. In animal models symptoms of inflammatory arthritis and autoimmune encephalomyelitis improved after treatment with NAMPT inhibitors [32,33]. From those reports, NAMPT inhibition is likely to hold potential for the treatment of inflammation-related disorders.

Low-grade inflammation is present in obesity and atopic diseases and an increase of NAMPT serum concentration in obese children and adolescents has been shown previously [8,34,35]. We, therefore, expected increased NAMPT levels for these groups. However, we did not find altered NAMPT concentrations in obese children, neither did we detect differences in NAMPT levels in patients with atopic diseases compared to healthy controls. We suppose that differences between the groups are smaller than we could detect in this analysis due to the limited number of participants in our study.

Regarding anthropometric data no relevant factors influencing NAMPT serum concentrations could be detected. In correlation analysis age was found to be negatively associated with NAMPT levels, which did, however, not withstand adjustment to height SDS and neutrophil count. In our cohort patients with acute infectious diseases were younger than the remaining cohort (because acute infections leading to hospitalization are more common in infants than in older children), which may have biased the univariate analysis. Our previous studies in healthy children did not show a correlation of NAMPT with age [11]. Looking into literature no significant relationship between NAMPT and age, gender, pubertal stage, height or circumferences of waist and hip had been evident in multivariate analysis [34]. Hence, no strong evidence for any independent anthropometric factor on NAMPT serum levels could be detected.

## Conclusion

In conclusion we report that NAMPT is a selective marker for acute and active chronic inflammation in children. NAMPT may hence also serve as an indicator for disease activity in chronic inflammatory diseases. The close relationship of systemic circulating NAMPT with leucocyte counts and release indicate that leucocytes, particularly neutrophils, are the source of inflammation associated NAMPT levels.

## Supporting information

S1 Table Multiple regression analysis in the cohort. (DOCX)
