# Supplementary material for: NAMPT serum levels are selectively elevated in acute infectious disease and in acute relapse of chronic inflammatory diseases in children
Source: PLoS One. 2017 Aug 24;12(8):e0183027. doi: 10.1371/journal.pone.0183027 (PMC5570332; doi:10.1371/journal.pone.0183027)
Supplement: S1 Table — (DOCX) [file pone.0183027.s001.docx]

| **Supplementary Table 1. Multiple regression analysis in the Cohort** | | | | | | |
| --- | --- | --- | --- | --- | --- | --- |
| **A.** Multiple regression analysis for the influence of anthropometric parameters on NAMPT serum levels in the control group | | | | | | |
| variables | | statistical model: multiple R^2^=0.55; n=14; *p*=0.09 | | | | |
| dependent | independent | step | parameter | Δr^2^ | b±S.E.M. | *p* |
| NAMPT* | BMI SDS, age, PH, gender, height SDS | 1 | Height SDS | 0.47 | 0.74±0.28 | **0.04** |
|  |  | 2 | PH | 0.08 | -0.29±0.28 | 0.34 |
| **B.** Multiple regression analysis for the influence of anthropometric parameters on NAMPT serum levels in all study participants | | | | | | |
| variables | | statistical model: multiple R^2^=0,065; n=102; *p*=0.09 | | | | |
| dependent | independent | step | parameter | Δr^2^ | b±S.E.M. | *p* |
| NAMPT* | BMI SDS, age, ph, gender, height SDS | 1 | Age | 0.05 | -0.2±0.12 | 0.066 |
|  |  | 2 | gender | 0.02 | 0.14±0.12 | 0.24 |
| S.E.M, standard error of the mean; BMI, body mass index; SDS, standard deviation score; PH, puberty status. Significant *p*-values are indicated in **bold**. *Values were log-transformed before analysis | | | | | | |
